# Turnover at the Top: The Digital Transformation and Dismissal of Chairman and CEO

**DOI:** 10.3389/fpsyg.2022.883192

**Published:** 2022-05-02

**Authors:** Jipeng Qi, Yaxian Zhou, Wendai Lv, Qiyue Du, Ran Liu, Caixing Liu

**Affiliations:** ^1^Center of Mergers and Acquisitions Research, School of Economics and Management, Beijing Jiaotong University, Beijing, China; ^2^School of Economics and Management, Beijing University of Chemical Technology, Beijing, China

**Keywords:** digital transformation, turnover, social capital, dynamic managerial capital theory, political connection, social networks

## Abstract

Companies increasingly implement digital transformation strategies to promote efficiency. Nevertheless, there are few concerns about employees’ acceptance of the changes, especially the executives’ adaptability, which is an important part of digital transformation strategy implementation. By utilizing the “searching-matching” in keywords of the annual reports of public listed companies in China, we measured the degree of corporate digital transformation to analysis its influence on the turnover rate of the Chairman and CEO. We found that digital transformation decreases the possibility of Chairman and CEO’s turnover. Derived from the dynamic managerial capital theory, we demonstrated that executives’ social network and political connections both have a moderate effect on the relationship between digital transformation and the turnover rate of executives. These findings will contribute to the digital transformation research by integrating with executives’ dynamic managerial capital which is attained through social networks and political connections.

## Introduction

Does digital transformation change the behavior of the Chairman and CEO? Prior research notes that digital transformation has become a strategic imperative on leadership agendas (Fitzgerald et al., 2014; Hess et al., 2016). Leveraging big data analytics and self-learning capabilities, digital technologies can track individuals’ activities at work, assess job performance, and create recommendations for modifications that can enhance the productivity of the employees (Tong et al., 2021). Consequently, scholars focus a great deal on the benefits of digital transformation to executives’ behaviors such as prediction and decision making (Agrawal et al., 2018; Lee, 2018; Iansiti and Lakhani, 2020; Luo et al., 2021). Nevertheless, there are rarely concerns about the effects of digital transformation that could possibly cause the executives’ turnover rate.

That the Chairman and CEO’s turnover might derive from digital transformation has constructed a unique internal and external environment for companies that challenge the leadership of executives. To implement a digital transformation, executives must adapt to diverse changes within the organization. These changes include the business model (Karimi and Walter, 2015), the allocation of resources and capabilities (Cha et al., 2015; Yeow et al., 2018), the reconfiguration of processes and structures (Resca et al., 2013), and digital culture (Llopis et al., 2004). Leadership alterations (Hansen and Sia, 2015; Singh and Hess, 2017) and recruitment of digital talents (Tong et al., 2021) are also given as requirements to the executives. For the external environment, society faces disputes of digital transformation, and a concern exists that the implementation of digital transformation, particularly with no transparent policy may cause distrust and harm morale (Premuzic et al., 2018; Cheatham et al., 2019). Thus, the implementation of digital transformation might amplify the requirements of executives’ leadership and increase the possibility of companies getting involved in the social controversy. Nonetheless, the use of big data and AI analytics help executives to comprehensively track their behavior and create personalized recommendations for job improvement (Colangelo, 2020; Heaven, 2020). Big data and AI analytics also assist the executive to handle a complex environment and make efficient decisions (Agrawal et al., 2018). When faced with drastic change, the advantages of digital technology may assist executives to improve their leadership and capabilities. Meanwhile, the successfully implementation of digital transformation has demonstrated the capability and leadership of both the Chairman and CEO. Dikolli et al. (2014) point out that an executive’s ability is connected to the length of their tenure. Consequently, the implementation of digital transformation benefits both executive’s tenure. Even so, beyond anecdotes and industrial reports, the influence of digital transformation implementation on the dismissal of executives has not been investigated adequately or in a systematic manner in academic literature. Therefore, there is a dearth of systematic understanding about whether and how executives adapt to the digital transformation and decrease the possible negative effects of digital transformation specifically for themselves.

Motivated by clarifying the possible effects of digital transformation on executives’ turnover, we analyzed the change in companies’ internal and external environments and in addition, went through the digital transformation effects on the executives’ turnover decisions. We established that digital transformation is negatively linked to executives’ turnover. This outcome supports Agrawal et al. (2018) proposal that digital technologies assist executives to deal with the multifaceted environment and make efficient decisions. Based on active managerial abilities, we also confirmed that diverse social capital moderates the connection between digital transformation and executives’ turnover. When there is a firm implementation of digital transformation, the executives’ social networks are negatively linked to their likelihood of dismissal. In fact, the executive political connection is also negatively associated with their probability of dismissal, when companies implement a digital transformation strategy.

This article aspires to make several contributions to the preceding research. First, to the best of our knowledge, this study is the first to investigate how digital transformation influences Chairman and CEO’s turnover through different mechanisms. Previous studies focus a great deal on the effects of individuals’ characteristics on executives’ turnover and ignore the fact that active environment changes can also cause such a turnover. Campbell et al. (2011) find that CEOs’ lack of optimism is also related to their dismissal. Dikolli et al. (2014) suggest that an executive’s ability is connected to the length of their tenure. Cheng and Leung (2016) find that executives’ political connections lead to a decrease in the frequency of their dismissal. Overall, our research contributes to the antecedent of turnover literature by setting up research scenarios of the digital transformation that might challenge executives’ managerial capabilities.

Second, this article contributes to the literature that documents the influence of social capitalism on executives’ turnover. For example, executives’ social capital improves managerial performance (Moran, 2005) and reduces the resistance of the company’s digital transformation strategy implementation. Even though executives’ politics may lead to them being entrenched, it can also benefit the transformation of strategy implementation and bring in additional government resources. The executives’ social capital also benefits the company to espouse the environmental changes by acquiring knowledge and integrating different resources. These advantages promote the firm’s competitive advantage. It also benefits the executives’ careers and decreases the possibility of turnover. More importantly, our methodology for measuring digital transformation complements an increasing body of literature that links to the digital transformation. In order to use text analysis to measure digital transformation, we developed a keyword list for the future. The keywords are derived from the business models of new digital technologies. The reports on the Work of the Chinese Government can also be used to find the new trend of the digital transformation and give out keywords.

## Theory and Hypotheses

The increase in digital transformation causes diversity in the workforce that has helped reshape the business environment throughout the last decade. The executive must have active managerial capabilities in order to adapt to the environmental changes in and out of the organization. Executive dominant logic refers to a company’s decisions to stand up in its business environment and discover what it should be doing (Prahalad and Bettis, 1986). This logic represents many executives’ views of the world (Kor and Mesko, 2013; Wang et al., 2022) and influences their decision of turnover. Anderson et al. (2018) introduced one cause for managerial departure, namely, the growth-induced type of changing business environment. The growth-induced turnover refers to the departure of CEOs that are motivated by the environment. Managers must possess suitable capital to lead the company in its present circumstances. The digital transformation of a new environment may also influence executive turnover. Meanwhile, executives may also use their dynamic managerial capital which is derived from social networking and political connections to mitigate the influence of the environment. Consequently, we additionally integrate the research of social capabilities and political connection with digital transformation to find out how executives adapt to the environmental changes and decrease the potential effects of the digital transformation.

### The Influence of Digital Transformation on Companies’ Internal and External Environment

The term “digital transformation” can be defined as “a process that aims to improve an entity by triggering significant changes to its properties through combinations of information computing, communication and connectivity technologies (i.e., digital technologies)” (Vial, 2019, p. 118). The extensive diffusion of digital technologies such as artificial intelligence (AI), and big data analytics have the potential to revolutionize changes in individuals’ behavior (Verhoef et al., 2021). The role of digital technologies in company management is increasingly important (Agrawal et al., 2018; Lee, 2018; Iansiti and Lakhani, 2020; Luo et al., 2021). Digital technologies recognize ways to boost production in the workflow (Marr, 2018; Heaven, 2020). Meanwhile, Bodrožić and Adler (2021) note that digital transformation revolutions appear within and between technology, organization, and public policy. Generally speaking, digital transformation has constructed a unique internal and external environment for companies from the change and interaction between individuals, organizations, technologies, and public policies.

The unique internal environment changed by digital transformation refers to a company’s management use of the digital technologies such as AI technologies which have superior data analytical skills compared with humans (Verma and Agrawal, 2016; Jarrahi, 2018). Digital technologies alter the company’s internal environment in two aspects. At first, digital technologies lie at the heart of the “information role” of executives which support executive monitoring of the dissemination of information to members of the firm (Mintzberg, 1990). The big data and AI analytics endorse executives and employees to enhance individual efficiency (Davenport and Ronanki, 2018; Luo et al., 2019; Thiel, 2019) and additionally increase the executives’ leadership. This can be viewed as a positive environment that is created by digital transformation. Second, digital transformation changes a variety of levels within the organization, such as core business (Karimi and Walter, 2015), resources (Cha et al., 2015; Yeow et al., 2018), and culture (Llopis et al., 2004). Digital transformation also causes the leadership of executives to improve (Hansen and Sia, 2015; Singh and Hess, 2017). Thus, the implementation of digital transformation has altered the internal environment and promoted the improvement of executive leadership.

The external environment changed by digital transformation refers to technology, organization, and public policy (Bodrožić and Adler, 2021). Digital transformation appears to be the latest one of five technological revolutions that were identified by Schumpeter-inspired scholars. Seen through the lens of Schumpeter (1942) theory of creative destruction, digital technologies will change the organization (e.g., Michelman, 2017; Tapscott and Tapscott, 2017) and public policy. Beyond the new industries, the traditional industries are unsuited to the revolution because of the innate organizational forms and public policies (Murray et al., 2021). The development of digital technologies is increasingly dominated by huge private companies (Benaich and Hogarth, 2021). Based on the advantages of technology, large private companies can expand faster than conventional industries. The competition in the external environment of companies is more drastic for dissimilar industries. Consequently, when facing the competition of the external environment, the implementation of digital transformation demonstrates the strategic awareness of executives.

Based on the hypothesis of growth-induced managerial turnover (Anderson et al., 2018), the replacement of top management is forced by drastic changes in the circumstances. When facing internal and external environment changes, the executive needs to have the ability to lead the company’s performance as expected in the present environment. A CEO may be replaced because of the ineffective formulation and implementation of strategies and policies (Dikolli et al., 2014). The firm’s digital transformation implementation reveals the effectiveness of executives’ digital awareness and capabilities. Due to the successful implementation of digital transformation, the Chairman and CEO will not be replaced, but will further lead the firm to better adapt to the present circumstances. Thus, we suggest that: -

*Hypothesis 1:* the firm’s digital transformation reduces the probability of executives’ turnover.

### The Effects of Executives’ Social Capital

Adner and Helfat (2003, p. 1012) Define dynamic managerial abilities as “the capabilities with which managers build, integrate, and reconfigure organizational resources and competencies.” These dynamic managerial abilities can attain the firm’s competitiveness by altering environmental conditions (e.g., Teece et al., 1997; Sirmon and Hitt, 2009; Lin and Wu, 2014). The role of the manager is to redefine competitiveness within a changing and volatile environment (Castanias and Helfat, 1991; Mahoney, 1995). Scholars point out that the managers with greater active managerial capabilities will be more beneficial for the competitive advantage of a company (Helfat and Martin, 2015). Prior studies have shown that an individual’s active managerial capabilities come from social capital (e.g., McDonald and Westphal, 2003). Consequently, social capital can help executives upgrade their digital awareness and abilities and additionally enhance their leadership to handle problems with the process of digital transformation. The digital transformation of a firm’s performance reveals information about executives’ abilities to create value for shareholders and is a benefit to their tenure.

Social capital comes from the information and trust embedded in social networks (Woolcock, 1998). Earlier research has established that social capital is positively linked to the career success (e.g., Meverson, 1994; Fernandez and Weinberg, 1997). Executives can attain resources from formal and informal networks (Geletkanycz and Hambrick, 1997). Conversation with diverse colleagues within and outside the organization influences the executive to elucidate what is attainable by the company (Nahapiet and Ghoshal, 1998). By providing knowledge, heuristics, and interpretive lenses, social network influences executives’ decision-making. Preceding studies have revealed that social networks are beneficial to individuals’ career success (Lin et al., 1981; Boxman et al., 1991; Meverson, 1994; Fernandez and Weinberg, 1997). Balsam et al. (2017) point out that an indirect association between CEOs and outside directors decrease the likelihood of CEOs’ turnover. Executives’ social network also enhances the acquirement of their knowledge and resources (Podolny and Page, 1998; Rogers, 2010) and adapts to the digital transformation. Therefore, executives with additional social networks can more easily deal with problems during the process of digital transformation due to their extra resources and knowledge acquisition capability. These managerial capabilities from social networks will further decrease the possibility of the executives’ turnover.

Overall, the social network connection with dismissal decreases when the company implements digital transformation strategies from two aspects. One aspect is the social network can assist executives to improve their leadership and capability when facing the company’s digital transformation and attain better performance which is a benefit to their tenure. The other aspect is the information and trust entrenched in social networks may help the company gain additional resources to deal with problems during the process of digital transformation and further decrease the likelihood of turnovers. When the company focuses more on digital transformation strategy, the executives’ social network will be more important to their leadership and resource acquirement and adapt to the organizational changes. These benefits will decrease the possibility of executives’ dismissal. We propose that: -

*Hypothesis 2:* The executives’ social network moderates the association between a company’s digital transformation and their turnovers. When the company implements digital transformation, the executive social network is negatively connected to its likelihood of dismissal.

In China, political connection as another kind of social capital plays a fundamental role. Due to the Chinese government’s crucial role in resource allocation, it is hard to ignore governmental effects on companies (Zhang and Shen, 2022). Companies with political associations may affect the allocation of government resources and could receive more subsidies (Chen et al., 2020; Yu et al., 2020). Executives’ links with government officials may gain assistance and support. Chan et al. (2012a,b) show that the Chinese firms with political links have lower investment restrictions. When digital transformation is implemented, the allocation of resources (Cha et al., 2015) could cause resource scarcity such as capital scarcity. The executives’ political associations may gain the government’s support and resource to alleviate the difficulties of scarcity. From the entrenchment viewpoint, the executives’ political association can enhance managerial power. The managerial power may become entrenched if their firms have no scarcity of political connections. Hence, when facing a drastic change in digital technologies, this managerial power is not only a benefit to the company’s digital transformation strategy implementation, but also a benefit for it to gain more resources. This political connection entrenched the executives in the organization and will decrease their turnover (Ding et al., 2015).

Overall, when the firm implements a digital transformation strategy, the political connection is linked to turnover decline from the entrenchment perspective. From the entrenchment perspective, the executives’ political connection increases managerial capital and benefits the digital transformation implementation. The managerial capital entrenched the executives and further decreased their turnover. With a more digital transformation strategy focus, the executives’ political association will be more important and further deeply entrenched in the organization. This entrenchment will decrease the likelihood of dismissal. We propose that: -

*Hypothesis 3:* The executives’ political connection can moderate the connection between companies’ digital transformation and their turnovers. When the company implements digital transformation, the executives’ political connection will be negatively associated with their likelihood of dismissal.

### Sample and Data Collection

To test our theory, we gathered a dataset containing executive turnover during 2013–2020. Our sample consists of all the publicly traded companies on the Shanghai Stock Exchange and Shenzhen Stock Exchange. We gathered data on executive turnover as well as individual and company-level data from the CSMAR database. We excluded firms in the financial industry. We additionally required that all observations had no missing data for the variables of interest. At last, our study was based on a sample of 4,895 individual-company-year observations. All continuous variables are winsorized at the top and bottom 1% level to evade excessive values.

## Measures

### Dependent Variables

There are dissimilar reasons for executive turnover in the CSMAR database. These reasons include internal job relocation, retirement, changes in controlling shareholders, health problems, corporate governance reforms, legal disputes, term expiry, resignation, personal reasons, completion of active duties, dismissal, and others. Subsequent to prior research, the turnover of executives was classified into active and inactive turnover (e.g., Parrino, 1997; Huson et al., 2004). Specifically, we identified term expiration, resignation and dismissal, and personal as active departures, and retirement, health issues, legal disputes, and others as involuntary departures. We did not include internal job relocation, changes in controlling shareholders, corporate governance reforms, or completion of active duties in our study. We measured executive turnover using a dummy variable, denoted as turnover, which equals one if the turnover event is an active turnover and zero otherwise.

### Independent Variables

Digital transformation. Earlier research about digital transformation focuses much more on the hypothetical framework (e.g., Bodrožić and Adler, 2021). This research has not mentioned the quantitative measure of digital transformation. Thus, the scholars focus much more on the effects of the specific technologies such as AI (e.g., Lee, 2018; Iansiti and Lakhani, 2020; Luo et al., 2021; Tong et al., 2021), blockchain (e.g., Murray et al., 2021; Verhoef et al., 2021), and robotic (e.g., Agrawal et al., 2018; Acemoglu and Restrepo, 2020). To measure the digital transformation, we constructed a keyword list about digital transformation and used the keywords to locate digital transformation strategy in the text of the companies’ annual reports. The independent variable digital transformation is a proxy variable measured by the keyword frequency count of the annual report of the simple companies in this research. We constructed two independent variables of digital transformation separately. One uses the keyword list to construct the variable of digital transformation for the major model. The other strictly uses the phrase “digital transformation” to construct the variable, which is used in the robust check.

#### Social Connection

We gathered details of the social networks from the CSMAR database between 2013 and 2020, which records executives and directors concurrently serving as directors in other companies. As the digital transformation is a revolution in society, the executives concurrently serving as directors in other companies may acquire more knowledge and experiences. We measured the social network as the entire number of executives concurrently serving as directors in other companies.

#### Political Connection

We also obtained data on the executives’ political associations from the CSMAR database. To measure the executive’s political associations, we constructed a dummy variable—political connection equals one if the executive has or previously had an official title, and zero otherwise (Giannetti et al., 2015). Subsequent to this earlier research, we set up a dummy variable of a political association, which is represented by the political association, if the executive has or is presently serving in the central and local governments, courts, or procuratorates at all levels, or has served as a deputy in the people’s congress at all levels and is a member of the Chinese People’s Political Consultative Conference, the political connection value is 1, and 0 otherwise.

### Control Variables

Subsequent to earlier literature (e.g., Liao et al., 2014; Cao et al., 2017), we used accounting information from CSMAR and a series of firm-level characteristics that can affect executives’ turnover. In particular, the control variables we used in the analysis include board size, firm size, ROA, leverage, firm age. Board size is the entire number of executives in the firm. Firm size is the natural logarithm of entire assets. The term return on assets (ROA) refers to a financial ratio that signifies how profitable a company is in connection to its entire assets. Leverage is the total liabilities divided by the total assets at the end of the year (Lyu and Wang, 2020). Firm age is the natural logarithm of the time between the initial creation of a firm and the current time. In addition, we also selected a series of individual-level characteristics of executives as control variables that could influence the executive turnover (Jenter and Kanaan, 2015; Renneboog and Zhao, 2020). We select the gender, turnover age, tenure, and salary, where turnover age is the age of turnover executives and tenure is the length of the executives’ tenure. The salary of the turnover executive is also controlled.

### Methods

We used the binary logistic model to discover experiential proof of the effects of a company’s digital transformation on executive turnover. First, we examined the impact of the company’s digital transformation and executive turnover. Then, we ran the model using the variable digital transformation (containing extended keywords) and then used the strict digital transformation (which only contained the keyword “digital transformation”) as the robustness test. Second, we explored whether social networks and political connections affected the connection between digital transformation and executives’ turnover. Robinson and Schumacher (2009) pointed out that uncentered variables have higher intercorrelation, thus higher collinearity, which is an important step when testing interaction effects. Therefore, we use the centered variables to test the moderating effects.

**FIGURE 1:**
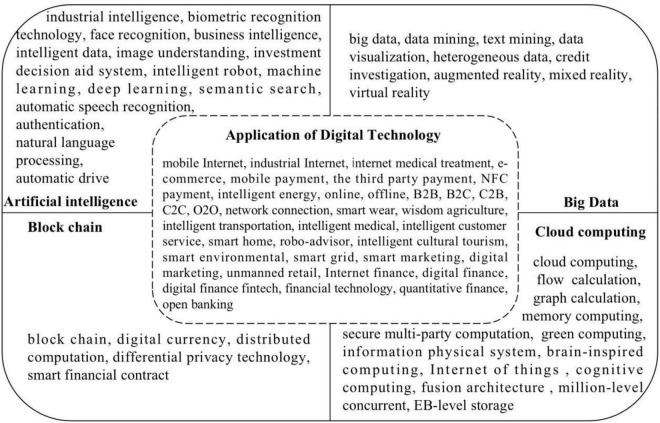
The keyword list of digital transformation.

We used text analysis to measure the independent variable digital transformation. Text analysis used keywords about digital transformation in the text of the annual report of listed enterprises. The reliability of this indicator is that the words in the annual report express the importance of an enterprise’s strategic orientation development. If specific keywords are expressed more often in the annual report, the company’s operation orientation and future development are focused much more on this field. Based on the Report on the Work of the Chinese Government in current years, we found the keywords about digital transformation and expanded the keyword database further. Finally, we formed a keyword atlas that is suited for this kind of research (see Figure 1). Use the Python crawlers, we extracted the keywords in the text of the annual reports. At last, we found the word frequency of every firm’s annual report and built the independent variable digital transformation. The number of keywords connected to the digital transformation was used to construct the variable of digital transformation. In the robustness test, we only used digital transformation as the keyword to construct the digital transformation to test the robustness model.

## Empirical Results

### Descriptive Statistics

Table 1 reveals the descriptive statistics and Pearson correlations for the main variables in the study. The mean (median) of turnover is 0.802, with a SD of 0.399, signifying that the majority of executives resigned voluntarily. The mean (median) value of the digital transformation is 0.541 and the mean (median) social network and political connection are 0.0462 and 0.206, which reveals that the social capital between the chairman and CEO is comparatively low in China. Individual-level and firm-level control variable results are also demonstrated in the table. No co-linearity issues existed among the variables in the sample, thus, the use of multiple regression analysis to test our hypotheses is supported.

**TABLE 1:** Descriptive statistics and Pearson correlation coefficients (*N* = 4,895).

	Variable	1	2	3	4	5	6	7	8	9	10	11	12	13
1	Turnover	1												
2	Digital transformation	–0.04***	1											
3	Social network	–0.02	0.014	1										
4	Political connection	–0.08***	–0.08	0.10***	1									
5	Gender	0.01	0.01	–0.02	–0.02	1								
6	Turnover age	–0.20***	0.02	0.03**	0.15***	0.06***	1							
7	Salary	0.03**	–0.03*	–0.09***	–0.01	–0.04***	–0.08***	1						
8	Tenure	–0.08***	0.01	0.03*	0.12***	0.02	0.35***	0.01	1					
9	Board size	–0.07***	0.08***	–0.01	0.05***	0.05***	0.12***	–0.34***	0.08***	1				
10	Firm size	–0.13***	0.10***	0.03**	0.03**	0.05***	0.15***	–0.22***	0.10***	0.37***	1			
11	ROA	–0.04***	0.02	0.03*	0.02	0.02	0.11***	–0.05***	0.07***	0.11***	0.13***	1		
12	Leverage	0.00	–0.02	0.00	–0.01	0.02	–0.05***	–0.10***	–0.03**	0.11***	0.34***	–0.38***	1	
13	Firm age	0.02	–0.04***	0.05***	–0.05***	0.01	–0.02	–0.05***	0.14***	–0.07***	0.19***	–0.18***	0.27***	1
Mean		0.802	0.541	0.0462	0.206	0.926	52.70	0.142	4.914	6.153	22.12	0.0155	0.475	2.281
Std.		0.399	1.706	0.210	0.404	0.261	7.494	0.0977	3.602	2.390	1.481	0.0999	0.236	0.799

*p < 0.1, **p < 0.05, ***p < 0.01.

### Hypothesis Testing

To additionally test our hypothesis, we carried out a binary logistic regression analysis. The result is revealed in Table 2. Model 1 is the base model. Hypothesis 1 suggested that the company’s digital transformation reduces the departure proclivity of the executive turnover. Model 2 supports hypothesis 1 (*p* < 0.05), as the coefficients of variables in the non-linear models do not represent the magnitude of the effects (Holburn and Zelner, 2010). The coefficient means that controlling for all other factors, the possibility of Y (executives’ turnover) is calculated by our model to increase by a factor of 0.955 when digital transformation is increased by one unit.

**TABLE 2:** Results of logit regression.

	(1)	(2)	(3)	(4)
Digital transformation		–0.0460**	–0.0455**	–0.0419*
		(–2.08)	(–2.02)	(–1.83)
Digital transformation* social network			–0.147*	
			(–1.84)	
Digital transformation* political connection				–0.108**
				(–2.26)
Social capital			–0.0395	
			(–0.27)	
Political connection				–0.298***
				(–3.30)
Gender	0.181	0.184	0.183	0.168
	(1.26)	(1.28)	(1.27)	(1.17)
Turnover age	–0.0655***	–0.0656***	–0.0656***	–0.0641***
	(–11.45)	(–11.45)	(–11.45)	(–11.12)
Salary	–0.348	–0.343	–0.328	–0.353
	(–0.82)	(–0.80)	(–0.77)	(–0.83)
Tenure	–0.00329	–0.00360	–0.00370	–0.000232
	(–0.30)	(–0.33)	(–0.34)	(–0.02)
Board size	–0.0119	–0.0102	–0.00992	–0.0103
	(–0.67)	(–0.57)	(–0.55)	(–0.58)
Firm size	–0.212***	–0.206***	–0.204***	–0.208***
	(–5.93)	(–5.75)	(–5.69)	(–5.76)
ROA	0.483	0.491	0.485	0.465
	(0.99)	(1.00)	(0.99)	(0.95)
Leverage	0.222	0.207	0.197	0.192
	(1.00)	(0.93)	(0.89)	(0.86)
Firm age	0.151***	0.151***	0.151***	0.140**
	(2.75)	(2.75)	(2.76)	(2.55)
Cons	8.985***	8.837***	8.769***	8.837***
	(10.14)	(9.95)	(9.86)	(9.91)
*N*	4,895	4,895	4,895	4,895
Year FE	Yes	Yes	Yes	Yes
Industry FE	Yes	Yes	Yes	Yes

*p < 0.1, **p < 0.05, ***p < 0.01.

**FIGURE 2:**
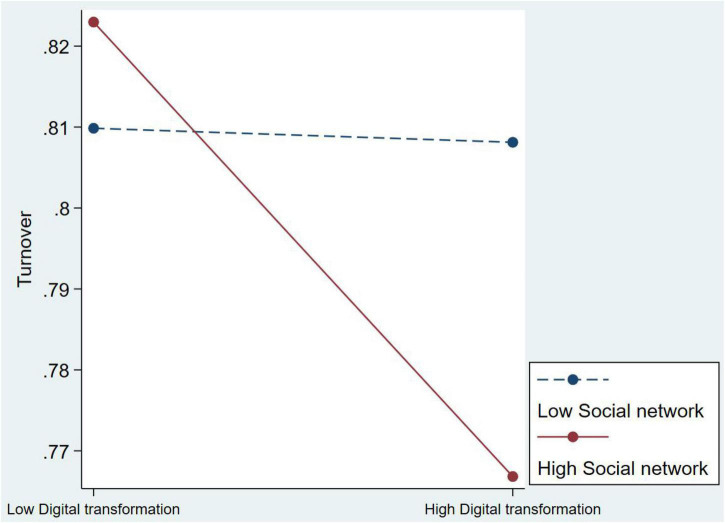
The moderating effect of social network on digital transformation for Chairman and CEO’ turnover.

**FIGURE 3:**
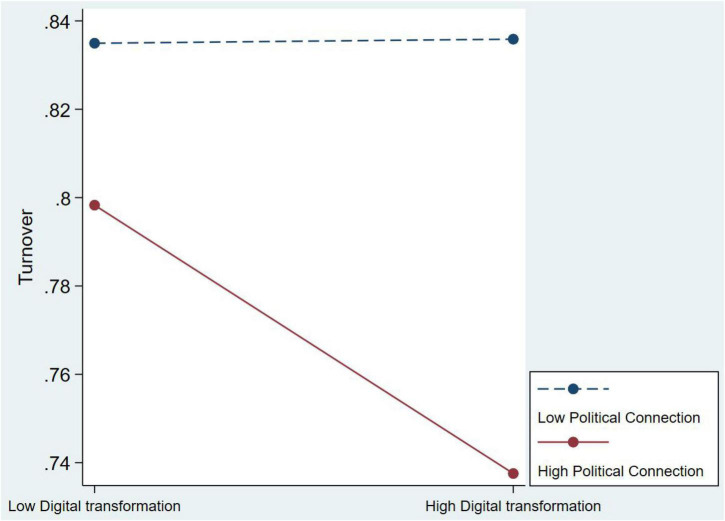
The moderating effect of political connection on digital transformation for Chairman and CEO’ turnover.

Hypotheses 2 and 3 are also supported. Model 3 reveals that the coefficient of interactions between digital transformation and social capital is –0.147 (*p* < 0.1). It calculates that when the social network is increased by one unit, the proclivity of executive turnover is 0.863. This signifies that when firms focus a lot on digital transformation, the executive with social capital is more inclined to stay. The slope test also support the hypothesis 2 (see Figure 2). The result of Model 4 is supported hypothesis 3; the coefficients of interactions of digital transformation and managerial social capital is –0.109 (*p* < 0.05). This signifies that when political connection increases by one unit, the proclivity of executive turnover is 0.897. The slope test also support the hypothesis 3 (see Figure 3).

### Robustness Tests

To investigate the sensitivity of our results to the model, we performed two methods of robustness tests. First, we used the keyword list to measure digital transformation which may expand the meanings of digital transformation which have not been defined clearly. Thus, we use “digital transformation” as the keyword to measure digital transformation. The results also support our hypothesis (Table 3). Second, we also tested a fixed-effect model, which created comparable results.

**TABLE 3:** The result of binary logistic model.

	(1)	(2)	(3)
Digital transformation	–0.0456**	–0.0451**	–0.0414*
	(–2.06)	(–2.00)	(–1.81)
Digital transformation* social network		–0.148*	
		(–1.85)	
Digital transformation* political connection			–0.109**
			(–2.27)
Social network		–0.0395	
		(–0.27)	
Political connection			–0.298***
			(–3.30)
Gender	0.184	0.183	0.168
	(1.28)	(1.27)	(1.17)
Turnover age	–0.0656***	–0.0656***	–0.0641***
	(–11.45)	(–11.45)	(–11.12)
Salary	–0.343	–0.328	–0.353
	(–0.80)	(–0.77)	(–0.83)
Tenure	–0.00359	–0.00370	–0.000228
	(–0.33)	(–0.34)	(–0.02)
Board size	–0.0102	–0.00993	–0.0104
	(–0.57)	(–0.55)	(–0.58)
Firm size	–0.206***	–0.205***	–0.208***
	(–5.75)	(–5.69)	(–5.77)
ROA	0.491	0.485	0.465
	(1.00)	(0.99)	(0.95)
Leverage	0.207	0.197	0.192
	(0.94)	(0.89)	(0.87)
Firm age	0.151***	0.151***	0.140**
	(2.75)	(2.76)	(2.55)
Cons	8.838***	8.771***	8.839***
	(9.95)	(9.86)	(9.91)
*N*	4,895	4,895	4,895
Year FE	Yes	Yes	Yes
Industry FE	Yes	Yes	Yes

*p < 0.1, **p < 0.05, ***p < 0.01.

## Conclusion

### Discussion

This study conducts a binary logistic model to formulate a meticulous inquiry into the association between digital transformation and executives’ turnover. The results support the findings that a company’s digital transformation reduces the likelihood of executive turnover. Moreover, from the managerial capital viewpoint, we discovered the moderating role of social capital. When a company focuses a great deal on digital transformation strategy, the executives with the additional social network are more liable to stay.

Political correlation as another kind of social capital plays a basic role in a firm implementation digital transformation strategy. When a company focuses a lot on digital transformation, the executives with the most political connections are less likely to leave. Conversely, when faced with digital transformation, the executives with more active managerial capabilities have less inclination to leave.

### Theoretical Implications

Our study makes several hypothetical contributions. First, we contributed to executives’ turnover literature by finding out the influence of the environment that might challenge executives’ managerial abilities. In previous literature, research on the executives’ turnover caused by digital technologies innovation environment changes is in short supply (Bodrožić and Adler, 2021). With a growing number of enterprises dedicated to implementing a digital strategy, the challenge for executives is becoming ever more complex. Consequently, our research on the antecedents of the executives’ turnover also extends the hypothetical picture of digital transformation research.

In addition, our research adds to emerging research on active managerial capital. Even though studies have revealed that the social capital is positively connected to career success (e.g., Meverson, 1994; Fernandez and Weinberg, 1997), facing digital transformation, the underlying role of executive’s social capital in their career is not definitely obvious. This study introduces social capital into our hypothetical model, in line with the concept drawn from research on executives’ turnover. From the viewpoint of active managerial capital (Adner and Helfat, 2003), our results show the moderating effect of social capital on the association between digital transformation and executives’ turnover. It indicates that facing the digital transformation, the executives’ social network, and political connections are both negatively connected to their likelihood of dismissal. On the one hand, the moderating model enriches the hypothetical research on active managerial capital; on the other hand, it also contributes to helping companies better retain executives. Furthermore, this article believes that the measure of digital transformation merits additional exploration.

### Practical Implications

The practical implications of our research contribute to the companies’ implementation of digital transformation strategies. First, this research can help companies to implement digital transformation strategies. The results show that companies’ focus on digital transformation decreases the proclivity of executives’ turnover. Considering the important role of executives in the planning and deployment of digital transformation (Johnson and Lederer, 2010), it is critical for companies to focus a great deal on the reasons for their turnover. Being aware of the importance of digital transformation, the companies’ ought to speed up the implementation of this strategy and actively adapt to the external environment. As a drastic change of the environment, the companies’ ought to accurately analyze the effect of digital transformation on the companies’ management system and initiative to change in order to retain the executives. Furthermore, companies could implement a digital transformation strategy by supplying leaders with the opportunity to practice their managerial ideas.

Second, our study shows that dynamic managerial capital plays a critical role in the decrease of executive turnover. This makes us aware that firms ought to attract executives with more active managerial capital because such executives would assist the company to attain its competencies by changing the environment (e.g., Sirmon and Hitt, 2009; Lin and Wu, 2014). Specifically, companies can actively attract executives with more social capital to enhance the digital transformation strategy implementation. In addition, by attracting executives with more political connections, companies could better adapt to the external environment changes especially in response to the needs of government digital transformation.

### Limitations and Future Research Directions

Even though the theory suggested in this study has been confirmed, the ensuing limitations remain. One is that we did not fully measure the digital transformation, which has certain limitations in the confirmation of causality. As digital transformation is an active process and technologies are changing quickly, future studies can measure digital transformation by adding new keywords. The new keywords could be new technology, new business model, and other new things.

Second, this study tests the moderator role from the active managerial capital. Even though the social capital and political capital are two crucial facets of the executives, other facets of active managerial capital may also influence the connection between digital transformation and executives’ turnover rate. Earlier research notes that managerial human capital plays a significant role in forming executives’ decisions (Kor and Mesko, 2013). When facing a drastic change environment, the executive with managerial human capital is more prone to leave for a new opportunity, particularly, if they already have digital awareness and capabilities. Thus, future research may incorporate managerial human capital into the research. Third, the effect of gender on the executives’ turnover rate was not seriously considered in this study. Studies have proven that males and females have a different tendency for turnover (Krishnan, 2009). In the future, gender should be seriously considered in the study of digital transformation and executives’ turnover rate.

At last, we discussed the connection between digital transformation and executives’ turnover rate with a binary logistic model. There could be other factors that may influence the association between digital transformation and executives’ turnover rates, such as company performance Just as the growth-induced managerial turnover (Anderson et al., 2018) depicts the company’s performance and plays an important role in CEO turnover. When facing performance pressure and digital transformation, executives decide on a different career. The consideration of a company’s performance offers a new direction for potential research.

## Data Availability Statement

The original contributions presented in the study are included in the article/supplementary material, further inquiries can be directed to the corresponding author/s.

## Author Contributions

JQ developed the theoretical framework and worked on literature review and manuscript writing. YZ and WL developed the theoretical framework and worked on data collection and analysis. WL, QD, and RL worked on literature review and data collection. RL and CL worked on data analysis and manuscript writing. All authors contributed to the article and approved the submitted version.

## Conflict of Interest

The authors declare that the research was conducted in the absence of any commercial or financial relationships that could be construed as a potential conflict of interest.

## Publisher’s Note

All claims expressed in this article are solely those of the authors and do not necessarily represent those of their affiliated organizations, or those of the publisher, the editors and the reviewers. Any product that may be evaluated in this article, or claim that may be made by its manufacturer, is not guaranteed or endorsed by the publisher.
